# Cell Adhesion and Cytotoxicity Assessment of Collagen-Coated Electrospun PCL Membranes Functionalized with Silver Nanoparticles

**DOI:** 10.3390/membranes16010017

**Published:** 2025-12-31

**Authors:** Chiara Goisis, Davide Porrelli, Gianluca Turco, Barbara Medagli, Giovanni Papa, Martin Iurilli

**Affiliations:** 1 Plastic and Reconstructive Surgery Unit, Cattinara Hospital, Strada di Fiume, 447, 34149 Trieste, Italymartin.iurilli@phd.units.it (M.I.); 2 Clinical Department of Medical, Surgical and Health Sciences, University of Trieste, Strada di Fiume, 447, 34149 Trieste, Italy; 3 Department of Life Sciences, University of Trieste, Via Alexander Fleming 31/B, 34127 Trieste, Italy; 4 Clinical Department of Medical, Surgical and Health Sciences, University of Trieste, Piazza Dell’Ospitale 1, 34129 Trieste, Italybmedagli@units.it (B.M.)

**Keywords:** cytotoxicity, cell adhesion, cell proliferation, electrospun nanofibers, polycaprolactone (PCL), collagen coating, silver nanoparticles (AgNPs), wound healing

## Abstract

Chronic and complex wounds require biomaterials that are both cytocompatible and antimicrobial. Herein, electrospun polycaprolactone (PCL) nanofiber membranes were coated with Type I collagen and functionalized with silver nanoparticles (AgNPs). The main objective was to assess fibroblast adhesion, proliferation, and cytotoxicity. Membrane morphology and surface characteristics were analyzed in a previous work by SEM, AFM, and wettability measurements, confirming the transformation from hydrophobic PCL to fully wettable collagen-coated surfaces. In this study, Murine 3T3 fibroblasts were cultured on PCL, PCL–Collagen, PCL–Collagen–Citrate, and PCL–Collagen–AgNPs membranes. Cellular activity was quantified using Alamar Blue assays at 24, 48, and 72 h, while cytotoxicity was determined by LDH release. Cellular viability and adhesion were studied using confocal microscopy. All membrane types supported fibroblast growth, with collagen-coated samples exhibiting the highest metabolic activity. AgNPs-functionalized membranes sustained overall cell viability above 90%, with cytotoxicity values of approximately 10% at 24 h and 20% at 48 h. Antimicrobial evaluations demonstrated complete inhibition of *Pseudomonas aeruginosa* and *vancomycin-resistant Enterococcus*, and partial inhibition of *Staphylococcus aureus*. These results indicate that collagen-coated, AgNPs-functionalized electrospun PCL membranes exhibit both high cytocompatibility and significant antimicrobial activity, supporting their potential as advanced wound-dressing materials.

## 1. Introduction

Chronic and infected wounds represent a persistent clinical challenge, characterized by delayed healing, recurrent infections, and high economic burden [1,2]. These lesions typically exhibit persistent inflammation, microbial colonization, and biofilm formation, which disrupt the delicate balance between tissue degradation and regeneration, ultimately hindering the restoration of functional skin architecture [3].

Conventional wound dressings generally act as passive protective barriers, providing moisture control and mechanical protection but failing to actively modulate the cellular microenvironment. Consequently, they often lack the biological cues required to support cell adhesion, migration, and proliferation. Advanced biomaterials have therefore emerged as promising alternatives, aiming to combine structural support with bioactive and antimicrobial properties. Among them, electrospun nanofiber membranes have gained increasing interest because their nanostructured architecture mimics the fibrillar organization of the extracellular matrix (ECM) and offers a high surface-to-volume ratio that can promote cell attachment and nutrient exchange [4,5].

Polycaprolactone (PCL) is one of the most extensively used polymers for electrospinning, thanks to its biocompatibility, biodegradability, and favorable mechanical properties [6]. However, its intrinsic hydrophobicity and lack of cell recognition motifs significantly limit cellular adhesion and spreading. To overcome these limitations, surface modification strategies have been developed, including plasma treatment, chemical grafting, and natural polymer coatings.

Among these, the application of Type I collagen represents an effective approach to introduce cell-adhesive domains, such as integrin-binding motifs, that facilitate fibroblast anchorage, cytoskeletal organization, and subsequent proliferation [7,8].

In parallel, infection control remains a critical aspect of wound management. Silver nanoparticles (AgNPs) are widely studied for their broad-spectrum antimicrobial efficacy and sustained release profile, which can efficiently suppress bacterial growth and biofilm formation. Nonetheless, AgNP incorporation into wound dressings requires careful dose optimization, as excessive ion release may impair cell viability and interfere with tissue regeneration [9,10].

In this study, electrospun PCL membranes were engineered with a dual functionalization strategy: a Type I collagen coating to enhance cellular interactions and the incorporation of AgNPs to provide antimicrobial activity. The main objective was to evaluate the cytocompatibility of these collagen-coated, AgNPs-functionalized PCL membranes, with particular attention to fibroblast adhesion, proliferation, metabolic activity, and potential cytotoxicity.

Building on our previous work [11], which established the synthesis, physicochemical characterization (SEM, AFM, wettability), and antimicrobial performance of collagen-coated PCL nanofiber membranes functionalized with AgNPs, the present study provides biological validation to demonstrate their suitability for wound-healing applications. Here, we shift the focus from material characterization to the systematic investigation of cell–material interactions. Direct-contact assays, including fibroblast adhesion, proliferation (Alamar Blue), cytotoxicity (LDH release), and confocal microscopy, were performed over 24–72 h to quantify cellular responses not previously reported. In parallel, quantitative analyses of collagen and silver release were conducted to correlate membrane composition and release kinetics with fibroblast activity. A key objective was to determine whether the retained silver content, approximately 0.55 μg per membrane, corresponding to a loading efficiency of ~78% and an effective surface density of ≈2.8 μg/cm^2^, preserves high cytocompatibility (>90% viability) while maintaining antimicrobial relevance against wound-associated pathogens. Collectively, these data provide comprehensive biological validation to support the translational potential of these multifunctional membranes for advanced wound-dressing applications.

## 2. Materials and Methods

### 2.1. Materials

PCL (Mw 80,000 Da) was chosen as the base polymer due to its advantageous mechanical and processing properties, which support scaffold formation and stability [12].

Type I bovine collagen was applied as a surface coating to specifically enhance cell adhesion, proliferation, and cytocompatibility, facilitating cellular interactions with the polymer.

Silver nanoparticles (AgNPs) were synthesized in-house from silver nitrate (AgNO_3_), using sodium citrate as a reducing agent. In this work, reagent concentrations were selected both to ensure antimicrobial activity and, primarily, to maintain fibroblast viability while minimizing oxidative stress. AgNPs’ antimicrobial properties also reduce contamination risks, which indirectly supports healthy cell expansion [13,14].

PCL was dissolved in dichloromethane (DCM) and dimethylformamide (DMF) as solvents [15], and all reagents (Merck, St. Louis, MO, USA) were of analytical grade.

PCL membranes were produced by electrospinning. PCL membranes were plasma-treated with an air plasma cleaner to make them hydrophobic and suitable for a collagen and silver coating.

AgNPs were synthesized using the Lee–Meisel citrate reduction method [15]. AgNPs were applied by drop-casting 4 μL of the AgNPs citrate suspension at 176 ng/μL onto each circular membrane (diameter 5 mm, area ≈ 0.196 cm^2^), corresponding to a total nominal silver amount of 704 ng per membrane. This yields a theoretical surface density of ≈3.6 μg/cm^2^ of Ag on the membrane before rinsing.

After coating and the standardized rinsing step to remove loosely bound nanoparticles, ICP-MS measurements of membranes indicated an average retained silver of ~0.55 μg per membrane, corresponding to a loading efficiency of about 78% and an effective surface density of ≈2.8 μg/cm^2^.

The AgNPs dose was rationally selected based on MIC ranges reported in the literature for planktonic bacteria (25–250 μg/mL) and on our previous work on the same PCL–collagen–AgNPs system, where similar Ag loadings provided antimicrobial activity with acceptable cytocompatibility. The standardized AgNPs’ Lee-Meisel synthesis and supporting literature provide a robust foundation for the expected AgNPs properties in our study [16,17,18,19].

### 2.2. Characterization of Nanofiber Membranes

SEM, AFM, and confocal microscopy were used in a previous work [11] to characterize fiber morphology, surface roughness, and collagen distribution.

Release tests to quantify the release of collagen and silver from nanofiber membranes were performed to study the interaction between the membranes and the wound environment. The Bradford assay was used to determine protein concentration in a solution. The linear concentration range is 0.1–1.4 mg/mL protein, using BSA (bovine serum albumin) as the standard protein. The assay is based on the interaction of Coomassie Brilliant Blue G-250 dye with proteins. The dye binds to the basic and hydrophobic groups of proteins, changing from a reddish-brown color (cationic form) to a deep blue (anionic form). This change can be measured spectrophotometrically at about 595 nm, providing a quantitative measure of the protein concentration in solution.

The results were compared with a calibration line based on a protein of known concentration, in this case, BSA (bovine serum albumin). Collagen release was studied using collagen-only, citrate-added, and silver-added membranes to evaluate possible interactions between these substances and protein release. The analysis was done at 24 h, 72 h, and 7 days to construct a release curve over time. The Bradford assay measures total protein content, so collagen release was inferred based on membrane composition. However, the structural integrity of the released collagen was not directly verified.

Silver release was assessed by immersing membranes (n = 3) in 5 mL PBS (pH 7.4, 37 °C) under gentle agitation (100 rpm). At 24 h, 72 h, and 7 days, 1 mL aliquots of the release medium were collected and replaced with fresh PBS. The aliquots were directly analyzed by ICP-MS for ^107^Ag (calibration range 0.1–100 μg/L, R^2^ = 0.999; LOD 0.05 μg/L). Preliminary blank and spike controls confirmed that the presence of citrate and collagen in solution did not produce detectable analytical interference in the silver measurements.

### 2.3. In Vitro Biological Evaluation: Adhesion and Proliferation

#### 2.3.1. Cell Culture and Experimental Design

Murine 3T3 fibroblasts were cultured in hg (high-glucose) DMEM (Dulbecco’s Modified Eagle Medium) with 10% FBS. After confluence, cells were seeded at 40,000 cells/mL in 500 μL into 24-well plates. Then, two sterile 5 mm membranes (PCL, PCL–collagen, PCL–collagen–citrate, or PCL–collagen–AgNPs) were placed in each well. Depending on the assay, cells were incubated with the membranes for 24, 48, or 72 h. AgNPs content was chosen to limit ROS-mediated toxicity while maintaining antimicrobial activity [19,20].

#### 2.3.2. Alamar Blue Proliferation Assay

Adhesion and proliferation on the membranes were further evaluated using Alamar Blue, a non-destructive viability assay. For the study, 48-well plates were used. A sterile 5 mm membrane was inserted into each well. After a 30 min soaking rest in hg DMEM, the membranes were wetted with 250 μL of hg DMEM containing 3T3 cells at 20,000 cells/mL. In this way, each well contained a 5 mm membrane covered with 5000 murine embryonic fibroblast cells.

Cell proliferation was analyzed at 24 h, 48 h, and 7 days. At the end of each interval, the media were removed, Alamar Blue diluted 30× was added to the wells, and the mixture was incubated for 4 h. After 4 h, the contents of each well were transferred to a dark plate for analysis with the Infinite M Nano+ fluorescence multimode microplate reader. Fluorescence was measured at λ_ex 560 nm and λ_em 590 nm using the Infinite M Nano+ microplate reader. This allowed the same cultures to be followed over time.

#### 2.3.3. Qualitative Microscopic Evaluation of Cell Morphology

To support the quantitative Alamar Blue and LDH assays, qualitative confocal microscopy was used to confirm cell adhesion, proliferation, morphology, and viability on nanofiber membranes containing AgNPs, a key element of the study.

To visualize cell distribution on the scaffolds via confocal microscopy, cells on the membranes were stained with fluorescent dyes to label the cytoskeleton and nucleus, enabling assessment of cellular morphology and health in the presence of the membrane. PCL, PCL–collagen, PCL–collagen–citrate, PCL–collagen, and AgNPs–functionalized membranes in citrate, after Alamar Blue proliferation assays, were fixed with 4% paraformaldehyde.

Following three washes with PBS, the samples were permeabilized for 30 min in PBS containing 0.1% Triton X-100, a non-ionic, non-denaturing detergent (PBS-T), to facilitate dye diffusion within cellular components. For cytoskeletal visualization, membranes were incubated in 200 μL of phalloidin diluted in PBS for 30 min. Nuclear staining was performed by soaking the membranes in 200 μL of DAPI (1000×; 4′,6-diamidino-2-phenylindole), a fluorescent dye that binds to DNA. Finally, membranes were mounted on slides for confocal microscopy analysis.

Distorted or abnormal cell morphologies indicate cytotoxic effects, whereas robust, well-spread cells are indicative of membrane biocompatibility.

### 2.4. In Vitro Biological Evaluation: Cytotoxicity

For the cytotoxicity assays, 3T3 fibroblasts were seeded at 40,000 cells/mL in 500 μL per well, corresponding to 20,000 cells per well. In each well, we placed two circular membranes with a diameter of 5 mm (area per disc ≈ 0.196 cm^2^), giving a total membrane area of ≈0.39 cm^2^ per well. This results in an initial density of ≈5.1 × 10^4^ cells/cm^2^ of membrane surface.

#### 2.4.1. LDH Cytotoxicity Assay

Cytotoxicity was assessed by measuring LDH release into the medium. Cells were cultured on the different membranes as above. Controls included cell lysates (total LDH), untreated cells, and medium-only wells. At 24 and 48 h, supernatants were collected and centrifuged. LDH activity was quantified colorimetrically using the Infinite M Nano+ (Tecan, Männedorf, Switzerland). Cytotoxicity was expressed relative to the total LDH control. It was corrected for spontaneous LDH release in cell-only wells.

#### 2.4.2. Alamar Blue Cytotoxicity Assay

As described previously in the adhesion and proliferation assays, the Alamar Blue assay relies on the reduction of resazurin, a non-toxic blue redox dye, to pink, fluorescent resorufin by metabolically active cells. Non-viable cells, lacking metabolic activity, are unable to reduce resazurin and thus do not emit fluorescence. The fluorescent signal intensity measured by a fluorimeter is directly proportional to the number of viable cells.

At each time point, the culture medium containing the membranes was removed, and Alamar Blue diluted 1:30 was added to the wells. The plates were incubated for 4 h, after which the contents of each well were transferred to a black plate for fluorescence measurement using the Infinite M Nano+ multimode microplate reader.

## 3. Results and Discussion

### 3.1. Membrane Characterization

SEM analysis of a previous study [11] confirmed uniform nanofiber morphology across all membranes, with diameters ranging from 350 to 550 nm. Collagen coating was evenly distributed. AgNPs appeared as discrete nanoscale clusters on fiber surfaces.

Compared with the original dataset, only essential characterization elements were retained, as the biological response constitutes the primary focus of this study. AFM measurements showed that collagen coating slightly smoothed the fiber topography, whereas AgNPs incorporation increased local roughness [11]. Both effects are known to influence early cell adhesion by altering the number of available integrin-binding sites [21].

**Figure 1:**
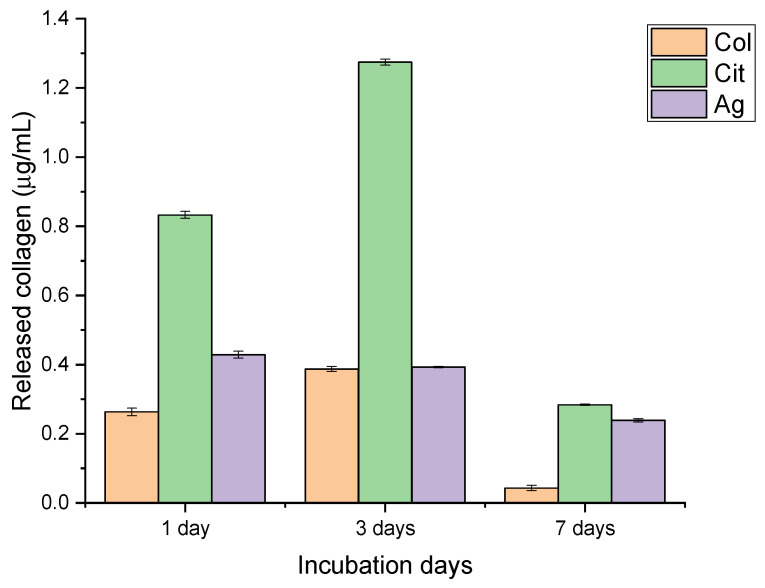
Collagen release from collagen-only (Col), citrate-containing (Cit), and silver-functionalized (Ag) nanofiber membranes after 1, 3, and 7 days of incubation. Data are presented as mean ± SD.

**Figure 2:**
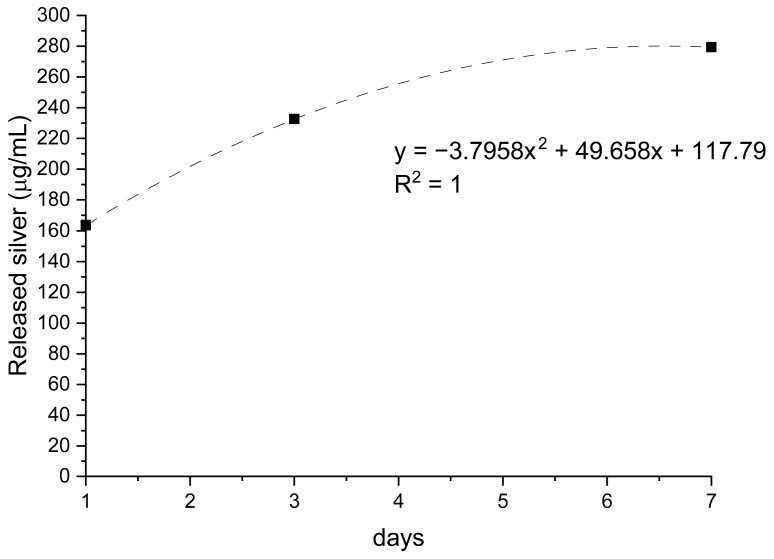
Silver release from AgNPs-functionalized membranes over 7 days of incubation. Data are expressed as µg/L, with trendline and corresponding regression equation (R^2^ = 1).

The release profiles of collagen and silver demonstrated clear compositional effects and distinct time-dependent trends (Figure 1 and Figure 2). Collagen-only membranes exhibited the highest protein release, with values of approximately 0.9 µg/mL after 24 h and a maximum of ~1.3 µg/mL at 72 h. The subsequent decrease observed at day 7 (~0.3 µg/mL) indicates depletion of loosely bound or surface-exposed collagen, supporting the interpretation of an initial burst-like wash-off rather than a sustained-release mechanism. No increase in absorbance at non-protein wavelengths was detected, excluding dye interference or degradation artifacts.

Citrate-containing membranes showed a consistently lower collagen release (≈0.6 µg/mL at both 24 and 72 h), followed by a modest reduction at day 7. This attenuated release suggests that citrate may stabilize collagen-surface interactions, limiting protein detachment. Although potential interference of citrate with the Bradford assay could be considered, the regular temporal trend and absence of spectral anomalies support the validity of the measurements.

Membranes incorporating AgNPs released the smallest amount of collagen throughout the study period (≈0.4–0.5 µg/mL at 24–72 h). This behavior is consistent with possible interactions between silver and protein functional groups that restrict collagen mobility. In parallel, the release of silver increased steadily from ~150 µg/L on day 1 to ~300 µg/L on day 7 (R^2^ = 1), indicating a controlled, gradual diffusion of AgNPs without an initial burst. This profile aligns with a progressive exposure of nanoparticles embedded within or beneath the collagen layer.

Taken together, these findings reveal that membrane composition significantly modulates release behavior. Collagen-only membranes maximize early protein availability; citrate-containing membranes offer an intermediate and more stable profile; and AgNPs-functionalized membranes combine limited protein release with sustained antimicrobial output. These characteristics are expected to influence fibroblast adhesion, proliferation, and cytocompatibility, as the balance between biochemical release and cellular response is central to membrane performance in wound healing applications. The distinct release profiles described here therefore provide a functional basis for interpreting the subsequent biological assays.

Collectively, these structural and surface properties provide the foundation for the biological responses discussed below, particularly cell adhesion, metabolic activity, and cytotoxicity.

### 3.2. In Vitro Biological Evaluation: Adhesion and Proliferation

#### 3.2.1. Alamar Blue Proliferation Assay

Alamar Blue assays at 24 and 48 h confirmed high metabolic activity and early proliferation on all membrane types. Collagen membranes produced the highest fluorescence, showing superior support for fibroblast adhesion and proliferation. Collagen-coated membranes had the highest cell viability, likely due to integrin-binding motifs and greater hydrophilicity, both of which are essential for cell adhesion and proliferation [11,22].

**Figure 3:**
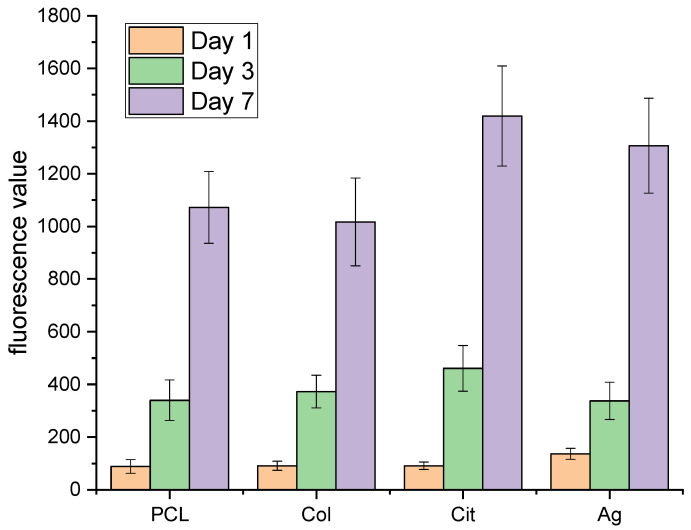
Proliferation of fibroblasts on PCL, PCL–collagen (Col), PCL–citrate (Cit), and PCL–Ag (Ag) membranes evaluated by Alamar Blue assay after 1, 3, and 7 days of culture. Data are expressed as relative fluorescence units (RFUs) and presented as mean ± SD.

AgNPs-functionalized membranes maintained fluorescence similar to that of collagen membranes, indicating that AgNPs do not impede early cell adhesion or proliferation. The non-destructive assay revealed sustained metabolic activity and potential for continued cell expansion (Figure 3). AgNPs-functionalized membranes maintained high biocompatibility, with only a slight reduction in Alamar Blue signal compared with PCL/Col membranes. This reflects careful tuning of AgNPs loading to preserve cell viability and proliferation, while also imparting antimicrobial activity. Notably, excessive AgNPs concentrations are linked with increased cytotoxicity via ROS generation [23]. This underscores the importance of precise dosage control.

#### 3.2.2. Qualitative Microscopic Evaluation of Cell Morphology

**Figure 4:**
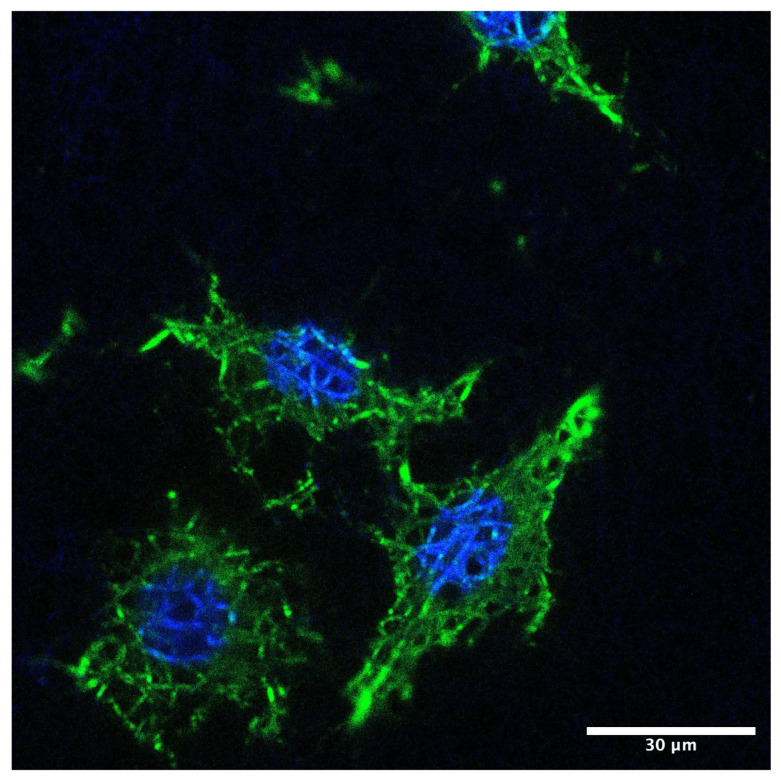
Confocal fluorescence micrograph of fibroblasts adhering to the nanofiber membrane. Actin cytoskeleton is visualized in green (FITC–phalloidin) and nuclei in blue (DAPI). Cells display elongated morphology and well-organized actin structures. Scale bar: 30 µm.

Confocal microscope images document the adhesion of 3T3 to nanofibers. Fibroblasts are morphologically elongated, well-spread, with good adhesion (Figure 4).

The synergism between collagen coating and the nanofibrous architecture creates an ECM-mimetic environment. This feature is essential for focal adhesion formation, cytoskeletal organization, and activation of signaling pathways governing proliferation and matrix deposition [24,25].

In conclusion, the data obtained indicate that the network structure simulates ECM, supporting cell adhesion and proliferation in all analyzed samples, even in the presence of AgNPs.

### 3.3. In Vitro Biological Evaluation: Cytotoxicity

#### 3.3.1. LDH Cytotoxicity Assay

Quantitative cytotoxicity was assessed using LDH assays. Control (untreated cells) showed 20% cytotoxicity at 24 and 10% 48 h. This elevated baseline LDH is attributable to the relatively high initial seeding density and rapid confluence of 3T3 fibroblasts, which are known to exhibit increased spontaneous LDH leakage under high-density conditions and after overnight attachment.

**Figure 5:**
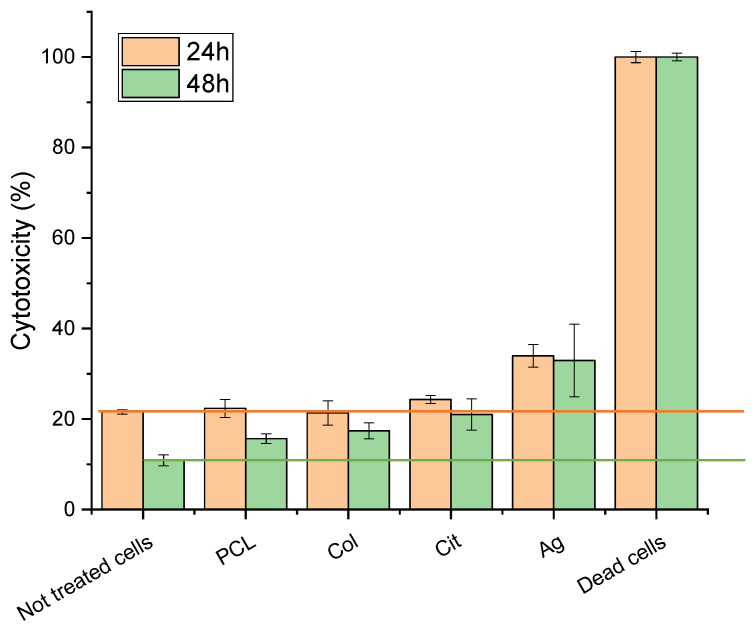
Cytotoxicity of fibroblasts cultured on PCL, PCL–Col, PCL–Col–Cit, and PCL–Col–Ag membranes measured by LDH assay at 24 h and 48 h. Results are reported as percentage cytotoxicity (mean ± SD). Dead cells were used as positive control.

All experimental group results were adjusted and presented as additional increases relative to the baseline. For PCL membranes functionalized with collagen and AgNPs, the incremental cytotoxicity was 10% at 24 h and 20% at 48 h. Total LDH release remained below 35% across all membranes, within accepted levels for wound-contact biomaterials (Figure 5). In this context, the data support low-to-moderate cytotoxicity for the tested membranes, especially in light of the consistently high cell viability observed in complementary assays.

Additional assays at an AgNPs concentration of 180 ng/μL confirmed effective antimicrobial activity and low-to-moderate cytotoxicity to fibroblasts.

At all evaluated time points, cell viability consistently remained above 90%.

#### 3.3.2. Alamar Blue Cytotoxicity Assay

**Figure 6:**
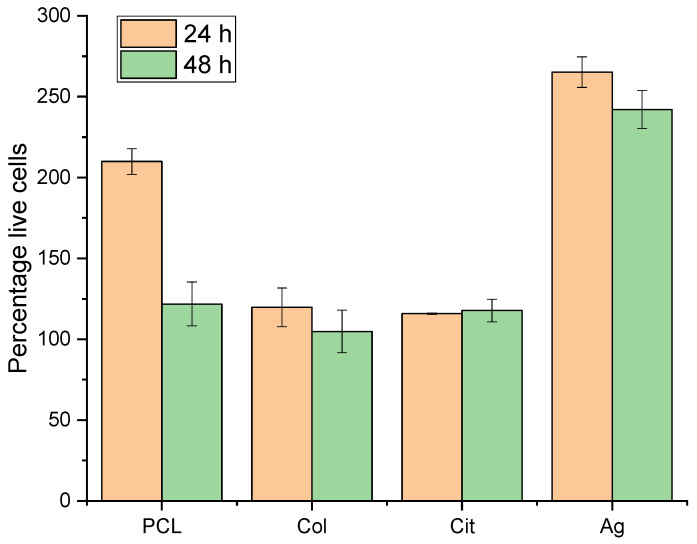
Cell viability on PCL, PCL–Col, PCL–Col–Cit and PCL–Col–Ag membranes assessed by Alamar Blue cytotoxicity assay at 24 h and 48 h. Data are expressed as percentage of viable cells (mean ± SD).

The Alamar Blue assay demonstrated that none of the materials that comprise the membranes studied in this thesis are cytotoxic. At both 24 h and 48 h of membrane contact, cultured 3T3 cells were unaffected by the membranes and did not die (Figure 6).

The Alamar Blue assay was employed to evaluate cellular responses during prolonged direct contact with the nanofiber membrane, accounting for potential leaching and surface-mediated effects over time. This direct-contact approach provides enhanced sensitivity and physiological relevance for assessing cell adhesion and proliferation on membrane surfaces, making it well-suited for membrane-focused studies.

The Alamar Blue assay presents several advantages, particularly pertinent to membrane research. Unlike the MTT assay, which necessitates cell lysis and yields only a terminal endpoint, Alamar Blue is non-toxic, enables real-time monitoring of cell viability and proliferation over extended durations without compromising cell integrity, and offers greater sensitivity in detecting subtle changes in cellular metabolic activity.

### 3.4. Clinical Implications and Future Directions

The biological outcomes observed in this study, such as strong fibroblast adhesion, sustained proliferation, and low-to-moderate cytotoxicity on collagen-coated PCL membranes with controlled AgNPs functionalization, highlight the potential relevance of these materials for chronic wound management. Clinically, combining high cytocompatibility with moderate antimicrobial protection may reduce infection risk. At the same time, the materials support tissue regeneration, which is essential for treating diabetic ulcers, venous leg ulcers, and pressure sores.

Building on these findings, future investigations should explore the in vivo wound-healing performance of PCL-Col-AgNPs membranes, including long-term biocompatibility, inflammatory response, and silver-release kinetics in physiological environments [26]. These studies will be essential for advancing the membranes toward clinical translation.

### 3.5. Study Limitations

Despite the promising results, this study presents several limitations that should be acknowledged.

All biological experiments were performed in vitro using murine fibroblasts, which may not fully replicate the complexity and cellular diversity of human wound environments.

Although silver’s standardized Lee-Meisel synthesis and literature [16,17,18] provide a robust foundation for AgNPs properties in our study, future work will include comprehensive direct characterization using TEM, UV-Vis, and zeta measurements.

Furthermore, although AgNPs functionalization was carefully controlled, the dynamics of silver release were not quantified in this study and should be investigated in future research to ensure long-term cytocompatibility and safety.

In addition, the long-term effects of AgNPs on inflammatory and regenerative responses were not evaluated, nor were potential cumulative toxic effects or the impact of prolonged silver release analyzed, highlighting the need for further studies on chronic exposure and tissue interaction.

A current limitation of our study is the lack of direct nanoscale mapping of coating thickness and chemical uniformity. To address this, we have initiated the use of infrared-scattering-type scanning near-field optical microscopy (IR s-SNOM) in ongoing and future work. This advanced technique will allow us to obtain quantitative, spatially resolved data on coating uniformity and crosslinking, enabling more robust correlations with biological and release profiles.

## 4. Conclusions

Electrospun PCL membranes coated with Type I collagen and functionalized with AgNPs were fabricated and characterized. Most importantly, they display a favorable cytocompatibility profile: strong fibroblast adhesion, high proliferation, and low-to-moderate cytotoxicity.

Alamar Blue assays, together with confocal microscopy images, demonstrated robust 3T3 fibroblast adhesion and proliferation on all collagen-containing membranes, including those with AgNPs. LDH release consistently indicated low cytotoxicity, with effective values around 10–20% at early time points and overall cell viability above 90%. These results confirm that controlled incorporation of AgNPs preserves, and may even enhance, fibroblast metabolic activity in vitro.

Collectively, these results demonstrate that PCL-Col-AgNPs membranes promote fibroblast adhesion and proliferation, minimize cytotoxicity, and deliver antimicrobial effects. Specifically, collagen coating enhances cell adhesion and proliferation, while a controlled AgNPs content provides antimicrobial activity without compromising fibroblast compatibility. These attributes make the membranes suitable for chronic wound applications and advanced wound dressings where cell compatibility is essential.

In summary, collagen-coated, AgNPs-functionalized electrospun PCL membranes provide maximal fibroblast compatibility, low cytotoxicity, and antimicrobial efficacy. Future research will focus on in vivo validation, long-term cytocompatibility, and the control of silver release for clinical translation.

## Data Availability

The raw data supporting the conclusions of this article will be made available by the authors upon request.
